# High-resolution analysis of multi-copy variant surface glycoprotein gene expression sites in African trypanosomes

**DOI:** 10.1186/s12864-016-3154-8

**Published:** 2016-10-18

**Authors:** Sebastian Hutchinson, Lucy Glover, David Horn

**Affiliations:** 1 Division of Biological Chemistry & Drug Discovery, School of Life Sciences, University of Dundee, Dow Street, Dundee, DD1 5EH UK; 2 Present address: Trypanosomes Molecular Biology, Institut Pasteur, 75015 Paris, France

**Keywords:** Allelic exclusion, Antigenic variation, Gene expression, RNA-seq, *Trypanosoma brucei*, VSG

## Abstract

**Background:**

African trypanosomes cause lethal diseases in humans and animals and escape host immune attack by switching the expression of Variant Surface Glycoprotein (VSG) genes. The expressed *VSGs* are located at the ends of telomeric, polycistronic transcription units known as *VSG* expression sites (*VSG*-ESs). Each cell has many *VSG*-ESs but only one is transcribed in bloodstream-form parasites and all of them are inactive upon transmission to the insect vector mid-gut; a subset of monocistronic metacyclic *VSG-*ESs are then activated in the insect salivary gland. Deep-sequence analyses have been informative but assigning sequences to individual *VSG*-ESs has been challenging because they each contain closely related expression-site associated genes, or *ESAGs,* thought to contribute to virulence.

**Results:**

We utilised ART, an *in silico* short read simulator to demonstrate the feasibility of accurately aligning reads to *VSG*-ESs. Then, using high-resolution transcriptomes from isogenic bloodstream and insect-stage Lister 427 *Trypanosoma brucei*, we uncover increased abundance in the insect mid-gut stage of mRNAs from metacyclic *VSG-*ESs and of mRNAs from the unusual *ESAG, ESAG10*. Further, we show that the silencing associated with allelic exclusion involves repression focussed at the ends of the *VSG*-ESs. We also use the approach to report relative fitness costs following *ESAG* RNAi from a genome-scale screen.

**Conclusions:**

By assigning sequences to individual *VSG*-ESs we provide new insights into *VSG*-ES transcription control, allelic exclusion and impacts on fitness. Thus, deeper insights into the expression and function of regulated multi-gene families are more accessible than previously anticipated.

**Electronic supplementary material:**

The online version of this article (doi:10.1186/s12864-016-3154-8) contains supplementary material, which is available to authorized users.

## Background

African trypanosomes are protozoan parasites that cause devastating diseases known as human African trypanosomiasis and a livestock disease known as nagana. These parasites are transmitted by the bite of an infected tsetse-fly, the distribution of which restricts the geographic spread of the disease. The parasite exists extracellularly, and is continually exposed to immune attack in the mammalian host [1]. To persist in the host bloodstream, the parasite has evolved a sophisticated strategy of antigenic variation and immune evasion. The trypanosome surface is coated in a dense layer of 10^7^ copies of a single variant surface glycoprotein (VSG) [2]. Switching of this VSG coat is central to adaptive immune evasion, and operates at a rate of approximately 10^−6^ per parasite cell division in culture [3]. In vivo, this leads to the recrudescent parasitaemia characteristic of *T. brucei* infection [1], where un-switched parasites are removed by antibody mediated killing.

*VSG* expression sites (*VSG*-ESs) are the key subtelomeric polycistronic units involved in antigenic variation in bloodstream African trypanosomes [4]. Understanding the expression and function of these units is critical to understanding virulence. *VSG*-ES transcription, mediated by RNA polymerase I, initiates at multiple *VSG*-ES promoters but is attenuated in all but one to prevent multi-*VSG* expression in individual cells [5]. The polycistronic *VSG*-ESs contain a number of Expression Site Associated Genes (*ESAGs*), several of which are of unknown function, but those that have been characterised are involved in nutrient acquisition; *ESAG6* and *ESAG7* [6], and innate immune evasion; *ESAG4* and *SRA* [7, 8]. In trypanosomes, maturation of mRNA from nascent transcripts occurs via the linked processes of *trans-*splicing, the addition of a 39-nt capped leader sequence, and poly-adenylation [9, 10]. RNA Pol-II transcribes the spliced leader from a repetitive array as a primary 135 b transcript [11], that is processed and 5′ capped before association with the spliceosome [12], which mediates *trans*-splicing to nascent transcripts [10].

Antigenic variation is specifically required for immune evasion in the bloodstream and, consistent with this, *VSG*-ESs are subject to developmental regulation. Upon parasite differentiation in the tsetse mid-gut, *VSG* transcription stops and the VSG coat is shed in the fly mid-gut, where recent evidence shows it interferes with fly innate immunity [13]. Procyclins, a family of repetitive proteins containing either EP or GPEET amino acid repeats, replace the VSG coat in the mid-gut [14]. Following migration to the fly salivary gland, a distinct sub-set of *VSGs* are expressed on the surface of metacyclic cells from monocistronic *VSG*-ESs, and are required for re-infection of the mammalian host [15, 16].

Next-generation sequencing (NGS) and RNA-seq approaches in particular, have been used in African trypanosomes to examine a range of features of genome organisation and gene expression, including developmentally regulated transcript expression [17], alternative splicing [18], control by RNA-binding proteins [19] and translation control [20, 21]. The approach has also been used to analyse relative expression levels for transcripts mapping to the active *VSG*-ES, revealing that most *ESAG* transcripts are present at 1–0.01 % the level of the active *VSG* transcript [17, 18].

NGS analysis of *VSG-*ESs presents several unique challenges. In particular, *VSG*-ESs are closely related [22] and, although increased mapping stringency can improve the alignment [23], the accuracy of assigning sequence-reads to the correct and specific sites has not been assessed in detail. Genes related to ESAGs (*GRESAGs*) are also found at non-telomeric locations; copies of *GRESAG4* are particularly prevalent and copies of *GRESAG2* are present at *procyclin* loci [24, 25]. In addition, *VSG*-ESs are under-represented in reference genome-sequence assemblies. Fortunately, the full set of *VSG*-ESs have been isolated and sequenced [22], and the subset of *VSG-*ESs expressed in the metacyclic stage has also been identified [15, 26], in the widely studied Lister 427 strain. However, developmental control of *VSG*-ESs has not yet been analysed in any detail in this strain.

We generated transcriptome data from sub-cloned populations of Lister 427 cells expressing a defined VSG (VSG-2) and from differentiated insect mid-gut stage cultures directly derived from those sub-clones. We then developed computational approaches to determine how accurately short reads derived from NGS can be aligned to *VSG*-ESs. We find that the differences between *VSG*-ESs are sufficient to allow 100-b reads to be accurately aligned to specific loci. Subsequent high-stringency mapping revealed a number of unanticipated features regarding *VSG*-ESs and their developmental control. High-stringency mapping was also applied to published NGS datasets. This revealed specific perturbations to *VSG*-ES transcriptomes following knockdown or over-expression of the allelic exclusion regulator VEX1 [27], and relative fitness costs following knockdown of individual *ESAGs* [28].

## Results

### Transcriptomes from isogenic bloodstream and insect-stage *T. brucei*

**Fig. 1:**
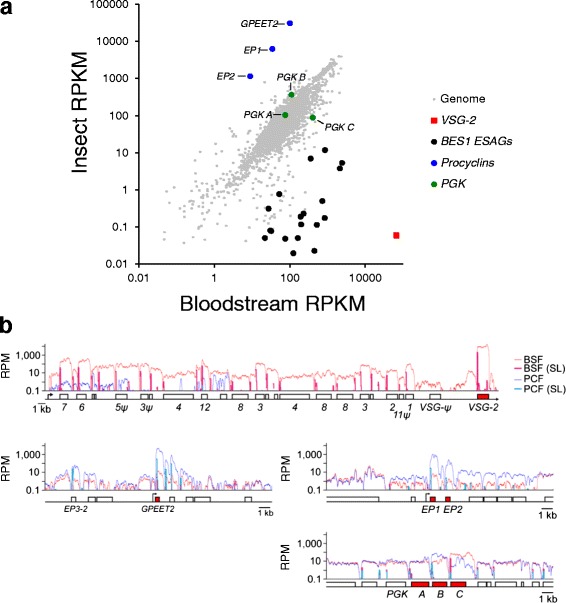
*T. brucei* transcriptomes from isogenic bloodstream and insect stage cultures. **a** The scatter plot shows RNA-seq data for bloodstream form and insect stage cultures. Values are calculated as RPKM (Reads Per Kilobase of transcript per Million mapped reads) and are averages for a pair of independent sub-clones. Selected developmentally regulated genes are highlighted. **b** Base-pair resolution plots of the active *VSG-*ES (top panel), *GPEET2* and *EP1/EP2* loci (middle panels) and the phosphoglycerate kinase locus (lower panel). Read counts are normalised using reads per million mapped (RPM) and trans-spliced reads are also indicated. Beneath each panel is a schematic map for each locus. Boxes, coding sequences; arrowsheads, Pol-I promoters

We derived transcriptomes from a pair of Lister 427 bloodstream form sub-clones expressing VSG-2 and from directly derived, differentiated, insect-stage cultures, separated from the bloodstream form sub-clones by only 10 days. Since the core, non sub-telomeric portion of the TREU-927 reference and Lister 427 genomes are closely related, sequence reads were aligned to the 11 megabase chromosomes from the TREU-927 genome [29], plus a non-redundant set of the 14 *VSG-*ESs [22] and 5 metacyclic *VSG*-ESs [15] from the Lister 427 strain. We aligned 26.4 and 26.5 million bloodstream form and 49.9 and 53.5 million insect-stage, 100 b, paired end reads using Bowtie2 [30], yielding approximately 180x genome coverage for bloodstream form and 350x for insect-stage transcriptomes. We calculated reads per kilobase of transcript per million mapped reads (RPKM), and differential expression using edgeR [31]; an additional table file shows these values (Additional file 1, ‘BSF v PCF mapq > 0’ tab). This revealed excellent reproducibility between clones, Pearson correlation coefficients of > 0.99 (Additional file 2), and robust developmental control of known bloodstream-form and insect-stage specific genes (Fig. 1a). For example, upon differentiation, the *EP* and *GPEET* procyclins increased 306 (EP1 and EP2 average) and 153-fold, respectively, while *VSG-2* decreased 1.2 × 10^6^ fold; BES1 *ESAG* transcripts were also decreased (Fig. 1a). In addition, phosphoglycerate kinase A (PGKA) displayed little change while PGKB increased (3.3-fold) and PGKC decreased (4.7-fold), as expected [32].

We next analysed reads from bloodstream and insect-stage cells that aligned to the active *VSG*-ES. A single base resolution BES1 plot (Fig. 1b) revealed a strikingly compact transcription-unit, incorporating little inter-transcript DNA sequence. Reads associated with a trans-spliced leader sequence, found associated with all trypanosomatid mRNAs [10], revealed trans-splicing at discrete points for each gene (Fig. 1b), as expected [33]. We observed multiple trans-splicing events within the *VSG* gene, but the dominant splice-site was used >1000-fold more frequently than other sites. As also expected, we see bloodstream-specific over-representation (266 fold on average) of transcripts for every *ESAG* present in the active *VSG*-ES (Fig. 1b), consistent with transcription attenuation following differentiation [34]. In the bloodstream-form, the *VSG* transcript itself is 141-fold more abundant than the mean value of the other *VSG*-ES-derived transcripts. We do see some isolated *ESAGs* that display higher expression relative to upstream *ESAGs* following differentiation to the insect stage but, rather than *VSG*-ES internal transcription initiation, this likely reflects incorrect assignment of reads from *GRESAGs* that are transcribed by RNA pol-II [24, 25]. Analysis of *procyclin* loci (also transcribed by RNA Pol-I) and the PGK locus (transcribed by RNA Pol-II) revealed similarly compact transcription units and the expected developmental controls (Fig. 1b). Thus, our RNA-seq datasets from isogenic bloodstream and insect-stage cultures are suitable for more detailed *VSG*-ES transcriptome analysis.

### ‘Short’ reads can be accurately assigned to *VSG*-ESs

We next considered the challenge of accurately assigning 100-b sequence reads from RNA-seq datasets to individual *VSG*-ESs. Analysis of *ESAG7* genes from the Lister 427 strain highlighted the challenge in terms of distinguishing among individual *ESAGs* (Additional file 3). In this case, a high level of identity was observed throughout the coding-sequence. There are differences however, which can be exploited.

**Fig. 2:**
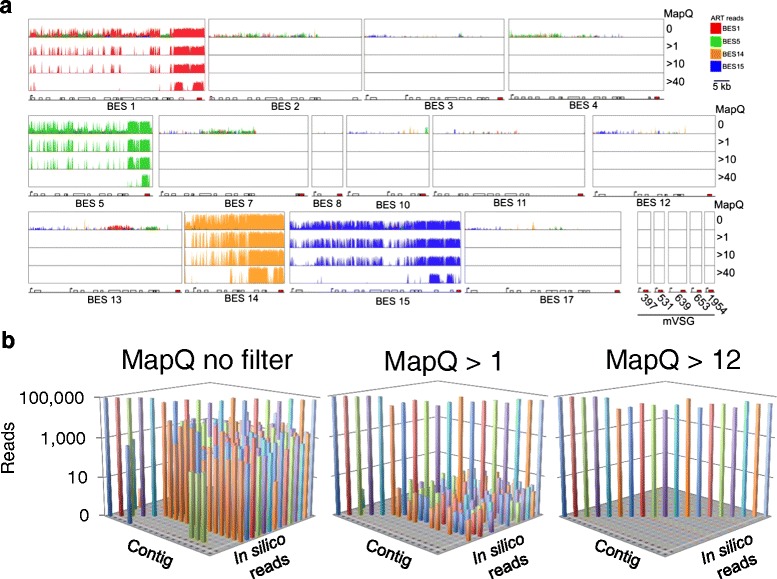
Simulations identity the optimal MapQ setting for accurate *VSG*-ES mapping. **a**. *In silico* reads generated from the ART program for a subset of *VSG*-ESs were aligned to the genome; only the *VSG*-ESs are shown on a linear scale. Varying the MapQ value (either no filter – 0, or incrementally > 1, > 10, > 40) to filter non-uniquely mapping reads improves the signal to noise ratio. mVSG, metacyclic *VSGs*. Beneath each panel is a schematic map for each locus. Boxes, coding sequences; arrowsheads, Pol-I promoters. Red reads BES1, Green reads BES5, Orange reads BES14, Blue reads BES15. **b** Analysis of all *VSG*-ESs using the ART method. Plots show reads derived from each contig, and aligned to the genome; only genes in *VSG*-ESs are shown. MapQ > 12 is the lowest value at which no noise is detected. Metacyclic *VSGs* are on the left

In order to determine the feasibility of accurate short read alignment to *VSG-*ESs, we used ART [35] to simulate the Illumina sequencing platform *in silico* and to generate 10^5^ single end, 100-b reads derived exclusively from individual *VSG*-ESs. We initially simulated Illumina sequencing runs of the sequence isolated from the active *VSG-*ES in our experimental strain and from three additional *VSG*-ESs (Fig. 2a). These reads were then aligned to the *VSG*-ES sequences using Bowtie2 [30], with the same settings used above. We observed reads aligning inappropriately to other *VSG*-ESs, however the signal to noise ratio could be increased significantly by filtering reads by the uniqueness (MapQ value) of the alignment, assigned to each read by Bowtie2 (Fig. 2a). This filters reads with a higher probability of being mis-aligned to the genome. We went on to repeat this *in silico* analysis with the entire set of assembled bloodstream and metacyclic *VSG*-ESs from the Lister 427 strain (Fig. 2b).

The analysis indicated that Bowtie2 aligns 75.5 % of *in silico* generated reads to the correct bloodstream *VSG*-ES with a MapQ > = 0 (98.7 % to metacyclic *VSG*-ESs) such that mis-aligned reads, as expected, can have a significant negative impact on transcriptome analysis. A MapQ value of > 1 removes 99.9 % of inappropriately aligned reads and retains 81 % of the signal while a MapQ > 12 eradicated all noise from the data and retained 65.7 % of the signal (Fig. 2b). We selected a MapQ cutoff > 1 as optimal for accurately assigning short sequence reads to individual *VSG*-ESs; the vast majority of *VSG*-ES associated genes retain short reads using this approach; an additional table file shows these values (Additional file 1, ‘BSF v PCF mapq > 1’ tab).

During this analysis, we also observed a distinct trend in the distribution of *in silico*-generated reads. Specifically, reads were more effectively retained closer to the telomere as we increased the uniqueness of alignment (Additional file 4A), indicating that sequences closer to telomeres are more divergent/unique. The alignment map for promoter-proximal *ESAG7* genes from Additional file 3 is also compared to an alignment map for the telomere-proximal *ESAG1* to further illustrate this point (Additional file 4B). Although a contested hypothesis [36], error-prone *VSG* gene-conversion was previously suggested as a mechanism contributing to antigenic variation [37, 38]. Our observation is consistent with this hypothesis when taken together with the inherent fragility of sub-telomeres and subsequent telomere-directed gene-conversion events [39].

### Differential controls affecting specific *ESAGs* and *VSGs*

**Fig. 3:**
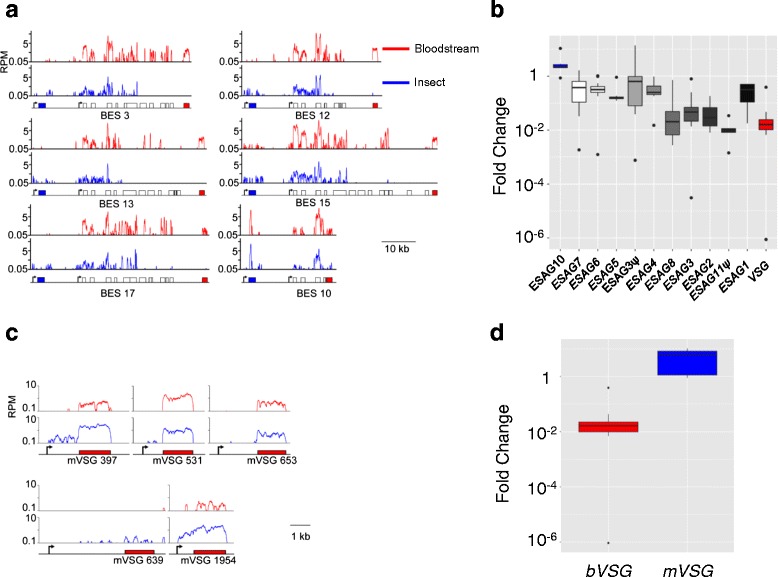
*VSG*-ES transcriptomes reveal unexpected features. **a** Base-pair resolution plots of six silent *VSG*-ESs containing *ESAG10* (blue boxes). Read counts were normalised using RPM. Beneath each panel is a schematic map for each locus. Boxes, coding sequences; arrowsheads, Pol-I promoters. Red lines, bloodstream-form; blue lines, insect-stage. **b** Box plot showing fold-change (log scale) from bloodstream to insect stage, of active and silent *ESAGs* grouped by type and position in *VSG*-ESs according to [22]. **c** Base-pair resolution plots of all five metacyclic-specific *VSG*-ESs. Red lines, bloodstream-form; blue lines, insect-stage. **d** Box plot showing fold-change (log scale) from bloodstream to insect stage, of bloodstream (*bVSG*) and metacyclic (*mVSG*) *VSGs*

Using a MapQ > 1, we filtered our RNA-seq data for high-confidence read alignments to *VSG*-ESs. The major developmental changes reported above (Fig. 1a) were also observed for this filtered dataset (Additional file 5). Filtering allowed us to assign reads to ‘silent’ *VSG*-ESs (Fig. 3a-b) and this revealed transcripts originating from the majority of *VSGs* in bloodstream-form cells (Additional file 5). This may reflect low-level expression of other *VSG*-ESs in cells expressing *VSG-2* or may equally reflect low-frequency activation of alternate *VSG*-ESs. Since our RNA samples were prepared using approximately 100 million cells and switching occurs in approximately one in every 10^6^ cells, we expect approximately 100 distinct switching events to be represented, although this does not so readily explain the expression of metacyclic *VSG* genes. As transcription is initiated at all *VSG*-ESs [5], proximity to the promoter may explain this low-level transcription of metacyclic *VSGs* that we observe in bloodstream form cells (Fig. 3c).

Reads mapping to ‘silent’ *VSG*-ESs are 2.4 × 10^4^-fold lower on average relative to those mapping to the active *VSG*-ES and are further reduced when cells differentiate to the insect stage (Fig. 3a). Again, we see some isolated *ESAGs* that display higher expression relative to upstream genes, likely reflecting reads from RNA pol-II transcribed *GRESAGs* [24, 25]. *VSG*-ESs share a generic structure, with similar *ESAGs* in similar positions. When grouped and represented according to their position, *VSG*-ES associated genes closer to telomeres display greater down-regulation in the insect-stage (Fig. 3b). For instance, average *VSG* expression level decreases 21-fold upon differentiation to insect stage cells, while ESAG7 expression decreased only 2 fold. In contrast to other *ESAGs*, four of six *ESAG10* genes were significantly (*p* < 0.05) upregulated (average 3.9 fold) in the insect-stage (Fig. 3a-b). This was unexpected since *ESAG* expression has been considered bloodstream stage-specific [40]. Thus, *ESAG10* may be an unconventional *ESAG* in terms of developmental expression-control.

Another unexpected observation was that, while *VSGs* in polycistronic *VSG*-ESs were down-regulated, three of the five *VSGs* located within the monocistronic metacyclic *VSG*-ESs were expressed at a significantly higher level (8.4 fold average, *p* < 10^−22^) in insect-stage cells (Fig. 3c-d, Additional file 5). *VSG* expression has been considered to be specific to the tsetse salivary gland stage and the bloodstream-stages, due to promoter control (the *VSG*-ES promoters active in bloodstream-form cells are distinct from the *VSG* promoters active in metacyclic cells) and stage-specific stabilisation of transcripts driven by a conserved element in the *VSG* mRNA 3′ untranslated sequence [41]. We speculate that the unexpected increase in expression of monocistronic metacyclic *VSG*s reflects progression to a metacyclic (−like) stage by small numbers of cells present in differentiated cultures. Indeed, increased expression of a single RNA binding-protein, RBP6, can trigger this progression through the life cycle [15]. Alternatively, sub-telomeric silencing may be less pronounced in insect-stage cells.

Our analysis of silent *VSG*-ESs allowed us to identify the *trans-*splicing sites for twelve *VSG*-ES linked *VSGs* and all five metacyclic *VSGs* from our RNA-seq data (Additional file 6A-B)*.* We counted polypyrimidine tract lengths for *VSG* splice sites and compared these to the genes in the RNA Pol-I transcribed *procyclin* loci, counting the number of consecutive pyrimidines and allowing for a single purine interruption. We found that the *VSG* genes are associated with significantly shorter polypyrimidine tracts (11.5 b, *n* = 17) compared to genes in the *procyclin* loci (19.0 b, *n* = 13, *p* < 4 × 10^−4^) or the 20 most abundant RNA Pol-II transcripts in our dataset (20.4 b, *n* = 20, *p* < 5 × 10^−4^) (Additional file 6C). Notably, *ESAG7* genes also possess shorter polypyrimidine tracts, suggesting that *VSG*-ES associated genes do not require extensive polypyrimidine tracts to form abundant mature messenger RNAs. Identification of splice-sites also allowed us to predict 5′-untranslated sequences and we note that there does not appear to be a consensus here; these sequences range in size from 15 to 91 b.

### Regulation of *VSG*-ES transcripts by VEX1

**Fig. 4:**
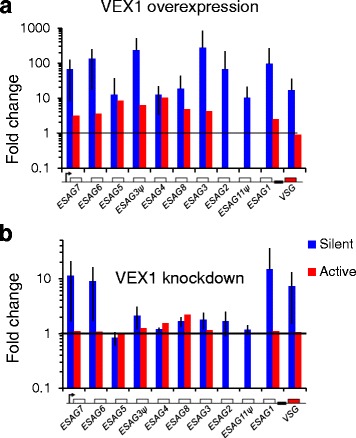
*VSG*-ES transcriptome analysis following VEX1 perturbation. **a** Average change of ‘generic’ [22] *ESAG* expression at either active (red) or silent (blue) *VSG*-ESs following overexpression of VEX1. **b** As in A but showing change in gene expression following knockdown of VEX1. Beneath each panel is a schematic map for each locus. Boxes, coding sequences; arrowsheads, Pol-I promoters. Black line crosses at 1 fold change (no change in expression)

VEX1 (VSG exclusion 1) is an allelic exclusion regulator required for the control of *VSG* gene expression [27]. In order to further our understanding of control by VEX1, we analysed the RNA-seq datasets generated following VEX1 overexpression (Fig. 4a) or RNAi knockdown (Fig. 4b), which both lead to increased expression of ‘silent’, ES-associated *VSG* genes [27]; an additional table file shows these values (Additional file 1, ‘VEX1’ tabs). High confidence (MapQ > 1) read alignments, of genes with > 10 reads when VEX1 was overexpressed, revealed an average of 5.4 fold more active *VSG*-ES *ESAG* transcripts, with 77 % of genes tested significantly increasing (> 3 fold change, *p* < 0.05) (Fig. 4a, Additional file 7, red bars), and an average 93-fold increase in silent *ESAG* transcripts, with 86 % of genes tested increasing significantly (> 3 fold change, *p* < 0.05) (Fig. 4a, Additional file 7, blue bars). These results are consistent with the previously reported positive control by ectopically expressed VEX1 [27].

We next analysed the RNA-seq data derived following VEX1 RNAi (Fig. 4b, Additional file 7). In this case, our analysis shows minimal impact at the active *VSG*-ES (Fig. 4b, red bars) but differential behaviour of the ‘silent’ *VSGs* and the *ESAGs* at either end of *VSG*-ESs relative to the centrally located *ESAGs*. Specifically, the promoter-adjacent *ESAG6* and *7* were increased 10-fold; 75 % of genes tested increasing significantly (> 3 fold, *p* < 0.05), while the telomere-proximal *VSG* and *ESAG1* increased 10 to 15-fold; 50 % of genes tested increasing significantly (> 3 fold, *p* < 0.05). This is in contrast to several centrally located *ESAGs,* which increased only 1.5 fold on average, with just 2 % of genes tested increasing significantly (> 3 fold, *p* < 0.05).

### Loss-of-fitness associated with *ESAG* knockdown

**Fig. 5:**
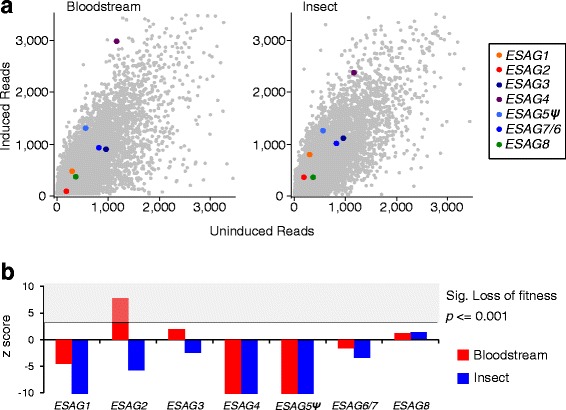
ESAG fitness profiles following high-throughput RNAi. **a** Scatter plots show genome wide quantification of fitness following *ESAG* knockdown as defined by RIT-seq in bloodstream (3 days of RNAi induction) and insect-stage cells. **b** Bar plots show z-scores reporting significant loss of fitness in bloodstream and insect RIT-seq experiments [28]

Our understanding of the functions of the *ESAGs* remains incomplete and, because of difficulties with accurate mapping as outlined above, the *ESAGs* were not analysed as part of prior high-throughput phenotyping analysis using RNA interference (RNAi) in *in vitro* culture [28]. We revisited these data and mapped RIT-seq reads (MapQ > 1) from bloodstream and insect stage cells to the *VSG*-ES active in the bloodstream-form (Fig. 5a); an additional table file shows these values (Additional file 1, ‘BES1 RIT-seq’ tab). Although the RNAi library used in the Lister 427 strain was derived from the TREU 927 strain, the closely related RNAi target fragments are still expected to mediate efficient *ESAG* knockdown [42]. Mapped reads were quantified and analysed relative to the uninduced control (Fig. 5b) revealing loss-of-fitness following *ESAG2*, *ESAG3* and *ESAG8* RNAi that achieved significance following *ESAG*2 RNAi; other *ESAGs* failed to register a loss-of-fitness. A previous study reported loss-of-fitness for every *ESAG* knockdown tested [43] but *ESAG2* also registered the greatest loss-of-fitness in that study. This phenotype may reflect the knockdown of RNA pol-II transcribed *GRESAG2* transcripts [24, 25] rather than *VSG*-ES associated *ESAG2* transcripts, however. None of the *ESAGs,* except for *ESAG8,* registered a loss-of-fitness in the insect-stage, consistent with developmental stage-specific *ESAG* expression*.* Notably, several *ESAGs* may be specifically required for host-parasite interactions, functions that are dispensable in *in vitro* culture.

## Discussion

Massive parallel NGS approaches have revolutionised the study of gene regulation and expression, producing data with an unrivalled depth [44], and have been applied with great success in African trypanosomes; see [17, 18, 33] for just a few examples. There are, however, a number of challenges associated with the analysis of subtelomeric sequences in a range of eukaryotes. These regions often incorporate highly repetitive and plastic components of the genome. This is particularly true of parasites, in which subtelomeric genes function in the processes of antigenic variation and immune evasion, such as malaria parasites [45] and African trypanosomes [4].

Analysis of these repetitive loci has proven challenging, a fact exemplified by our *in silico* analyses. Indeed, we note the use of orthogonal methods such as qRT-PCR and genetic tagging of *VSG*-ESs [46, 47], despite the availability of NGS approaches and datasets. We now report high-coverage transcriptomes from isogenic *T. brucei* cultures and from two major life cycle stages, namely the mammal-infective bloodstream form and the tsetse fly mid-gut stage, with a focus on the regulation of *VSG*-ES transcription. Simulated Illumina sequence data allowed us to gauge an appropriate filter that maximises the signal-to-noise ratio of sequence alignments at these loci. We find the expected extreme developmental regulation of *VSG* and *EP/GPEET* surface antigen genes and *VSG*-ES attenuation in insect stage cells but also, using high-stringency mapping, uncover additional and unexpected features.

Previous reports indicate that alignment of short reads to *VSG*-ESs can be problematic due to the similarity between these loci [22, 46, 48]. Our analyses show that an average of 24.5 % of *VSG*-ES derived reads are typically incorrectly aligned to *VSG*-ESs, and filtering reads with a MapQ value > 1 greatly reduces mis-mapping. Further analyses of *VSG*-ESs suggest that this is particularly useful for the closely related *ESAG* sequences. Improvements in sequencing technologies, such as quality (our sequence data has mean per-base quality scores > 34) and read-length now facilitate accurate high-stringency mapping. Specifically, we believe that 100–150 b reads that are commonly produced by current Illumina technologies incorporate sufficient SNPs to allow specific assignment to individual *VSG*-ESs.

In bloodstream cells, monoallelic expression ensures that a single subtelomere is productively transcribed [1]. However, in mid-gut stage cells, *VSG*-ESs are silenced [34]. In our populations, we find that transcripts for twelve silent *VSG*-ES linked and all five metacyclic *VSG*s are detectable, although at a level approximately 26,000 times lower than the active *VSG*; illustrating the impressive dynamic range of RNA-seq. In addition, our analysis reveals that *ESAG10* mRNAs, encoding putative folate transporters [49], are more abundant in the mid-gut stage cultures than the bloodstream form. This surprising finding is suggestive of less effective silencing in the insect-stage of *ESAG10*-associated *VSG*-ES promoters relative to the almost identical *VSG*-ES promoters located downstream [22]. Alternatively, *ESAG10* transcripts may display increased stability in insect-stage cells. Notably, additional RNA pol-II transcribed genes on chromosome 8 (Tb927.8.3620, 3630 and 3650) encode folate transporters and whether the *ESAG10*-associated promoters or transcripts are ‘activated’ at any point in the life cycle remains unknown.

High-stringency mapping using transcriptomic datasets derived following knockdown of the allelic exclusion regulator, VEX1 [27], revealed derepression of promoter and telomere proximal *VSG*-ES genes. In another study, ectopic overexpression of a second *VSG* gene resulted in *VSG*-ES silencing spreading from the telomere towards the promoter in a disruptor of telomeric silencing 1B (DOT1B) dependent manner [43]. Our analysis indicates that VEX1-mediated silencing is directed at the telomeric *VSG* and *ESAG1* genes, and at the *VSG*-ES promoter-adjacent *ESAG6* and *ESAG7* genes. This is in contrast to VEX1 overexpression, which upregulates all silent *ESAGs* and *VSG*s. Thus, our current data indicate that VEX1-mediated silencing primarily affects the ends of silent *VSG*-ESs, suggesting that subtelomere conformation may be important in the control of these genes. Finally, high-stringency phenotyping data confirm *(GR)ESAG2* as the *ESAG* associated with the greatest fitness cost when knocked-down in *in vitro* culture.

## Conclusions

By distinguishing between closely related transcription units, we have been able to enhance our understanding of the behaviour of *VSG*-ESs in terms of *VSG* silencing, developmental regulation and contributions to fitness in culture. NGS approaches, coupled to high-stringency mapping, such as RNA-seq, ChIP-seq, RIT-seq and the growing list of ‘seq’ technologies will undoubtedly improve our understanding of the organisation and expression of these virulence gene loci and indeed closely related gene families in a range of other organisms.

## Methods

### T. brucei

Two subclones of wild type bloodstream-form Lister 427 strain *T. brucei* expressing VSG-2 (VSG-221, Mitat1.2) were differentiated to insect mid-gut stage cells as previously described [50]. Briefly, cells were collected by centrifugation and resuspended in differentiation medium (DTM) [50] supplemented with 3 mM citrate and 3 mM cis-aconitate and maintained for 10 days at 27 °C, 0 % CO_2_ (ambient).

### RNA-seq

For RNA extraction, 5 × 10^7^ cells were collected and RNA prepared using the Qaigen RNeasy kit, according to the manufacturer’s instructions. Poly-A+ RNA was enriched using oligo-dT beads, and reverse transcribed. Second strand synthesis was randomly primed. Sequencing was performed on the HiSeq platform (Illumina) at the University of Dundee generating 100-b paired-end reads. This yielded insect-stage RNA-seq data, using identical processing, that were only 10-days removed from our bloodstream-form RNA-seq data [27].

In order to align reads we generated a hybrid genome assembly consisting of the 11 megabase chromosomes from the *T. brucei* 927 reference genome [29], the non-redundant set of 14 bloodstream expression sites [22] and the 5 metacyclic expression sites from our Lister 427 strain [15, 26]. Read alignment was performed using Bowtie2 [30] as previously described [27] using the parameters --very-sensitive --no-discordant. Approximately 25 million bloodstream-form and 50 million insect stage reads were aligned for each clone. Alignment files were manipulated using SAMtools [51], and visualized in the Artemis genome browser [52]. Single base resolution plots were generated using the pysam API (https://github.com/pysam-developers/pysam) in an in-house script that filters reads based on alignment quality (MapQ) and corrects for library size (available on request). Trans-spliced reads were extracted using a previously published script [53], using the partial spliced leader sequence ‘TCTGTACTATATTG’ and it’s reverse complement to search. This is the shortest sequence that returns only spliced leader sequences following BLAST search of the TREU-927 genome sequence on TriTrypDB. Differential expression analysis was performed with edgeR [31] as previously described [27]. When analysing VEX1 perturbation, we excluded genes with <10 reads averaged across replicates in both uninducing or inducing conditions.

### ART simulation

ART is a software package that simulates next-generation sequencing runs using empirical error models utilized by the 1000 genomes project [35]. Illumina sequencing runs were simulated for all 19 of the Lister 427 *VSG*-ES contigs in the hybrid genome using the parameters art_illumina -i contigX.fa -len 100 -ss MS -c 100000. This produced 10^5^ single-end reads for each contig; as the longest contig is 59,781 bp, this provided coverage of every base in each *VSG*-ES. These *in silico* reads were then aligned back to the complete genome with Bowtie2 using the parameters --very-sensitive [30]. Alignment files were manipulated using SAMtools [51]. Read counts were generated using the Artemis genome browser [52].

### Sequence and data analysis

Clustal alignment analysis and visualisation of *ESAG7* sequences was performed using CLC workbench using settings: gap open cost = 0.0, gap extension cost = 0.0, end gap cost = free, alignment mode = very accurate, redo alignments = no, use fixedpoints = yes. A non-redundant gene list was from [28] and *VSG*-ES sequences [22] were retrieved from TriTrypDB. ‘Generic’ *ESAG* lists are derived from [22]; *ESAG*s from each *VSG-*ES were compiled based on relative position within each *VSG*-ES.

## Additional files

Additional file 1: Table containing RNA-seq and RIT-seq data. (Excel 7530 kb)
Additional file 2: Comparison of RNA-seq replicates. Scatter plots of bloodstream-form and insect-stage RPKM values for each condition. Data used to derive plots in Fig. 1 (MapQ > =0). (PDF 28 kb)
Additional file 3:
*ESAG7* sequences are highly similar between *VSG*-ESs. Clustal alignment between *ESAG7* nucleotide sequence in BES1 (Tb429.BES40.2) and other *ESAG7* sequences. BES2, 4 and 8 have multiple *ESAG7* genes. (PDF 238 kb)
Additional file 4: Divergence increases towards the telomere. A. RPKM values for each *ESAG* and *VSG* in the non-redundant set plotted against the distance from the start-codon to the telomere. B. *ESAG7* Clustal alignment plot from Additional file 3 compared to ESAG1 Clustal alignment plot. (PDF 169 kb)
Additional file 5: High stringency RNA-seq analysis. Scatter plot of RPKM values of each condition with MapQ value > 1. Values are calculated as RPKM (Reads Per Kilobase of transcript per Million mapped reads) and are averages for a pair of independent sub-clones. Selected developmentally regulated genes are highlighted. Metacyclic (*mVSG*) and bloodstream (*bVSG*) specific *VSGs* are also shown. (PDF 23 kb)
Additional file 6:
*VSG* genes have significantly shorter polypyrimidine tracts upstream. A. Logo plots of the 17 *VSG trans-*splice sites identified from our data set (top) and genes in the RNA Pol-I transcribed *procyclin* loci. Pyrimidines, red; purines, grey. B. Individual *trans*-slice sites from beta-tubulin (top), bloodstream *VSG*-ES linked *VSGs* and metacyclic *VSGs.* Pyrimidines, red; purines, grey. C. Bar charts show average polypyrimidine tract length comparisons over several gene groups. ***, *P* <0.0005. (PDF 1442 kb)
Additional file 7: Individual *VSG-*ES transcriptome analysis following VEX1 perturbation. Bar-charts show fold-changes for *ESAG* expression in each *VSG*-ES following either VEX1 knockdown (blue bars) or overexpression (red bars). (PDF 32 kb)

## Abbreviations

BES: Bloodstream expression site
BSF: Bloodstream form
bVSG: Bloodstream VSG
ChIP-seq: Chromatin immunoprecipitation sequencing
DOT1B: Disruptor of telomeric silencing 1B
DTM: Differentiation trypanosome medium
*ESAG*: Expression-site associated gene
*GRESAG*: Gene related to *ESAG*
mVSG: Metacyclic VSG
NGS: Next-generation sequencing
PCF: Procyclic (insect) form
PGK: Phosphoglycerate kinase
qRT-PCR: Quantitative reverse transcription polymerase chain reaction
RBP: RNA binding-protein
RIT-seq: RNA interference target sequencing
RNA Pol: RNA polymerase
RNAi: RNA interference
RNA-seq: RNA sequencing
RPKM: Reads per kilobase of transcript per million mapped reads
RPM: Reads per million mapped
SRA: Serum resistance-associated
TREU: Trypanosomiasis research Edinburgh University
VEX1: VSG exclusion 1
VSG: Variant surface glycoprotein
*VSG*-ES: *VSG* expression site
