# Supplementary material for: High-resolution analysis of multi-copy variant surface glycoprotein gene expression sites in African trypanosomes
Source: BMC Genomics. 2016 Oct 18;17:806. doi: 10.1186/s12864-016-3154-8 (PMC5070307; doi:10.1186/s12864-016-3154-8)
Supplement: Additional file 2: — Comparison of RNA-seq replicates. Scatter plots of bloodstream-form and insect-stage RPKM values for each condition. Data used to derive plots in Fig. 1 (MapQ > =0). (PDF 28 kb) [file 12864_2016_3154_MOESM2_ESM.pdf]

Data Replicates

Bloodstream replicate Pearson = 0.999

Insect replicate Pearson = 0.998

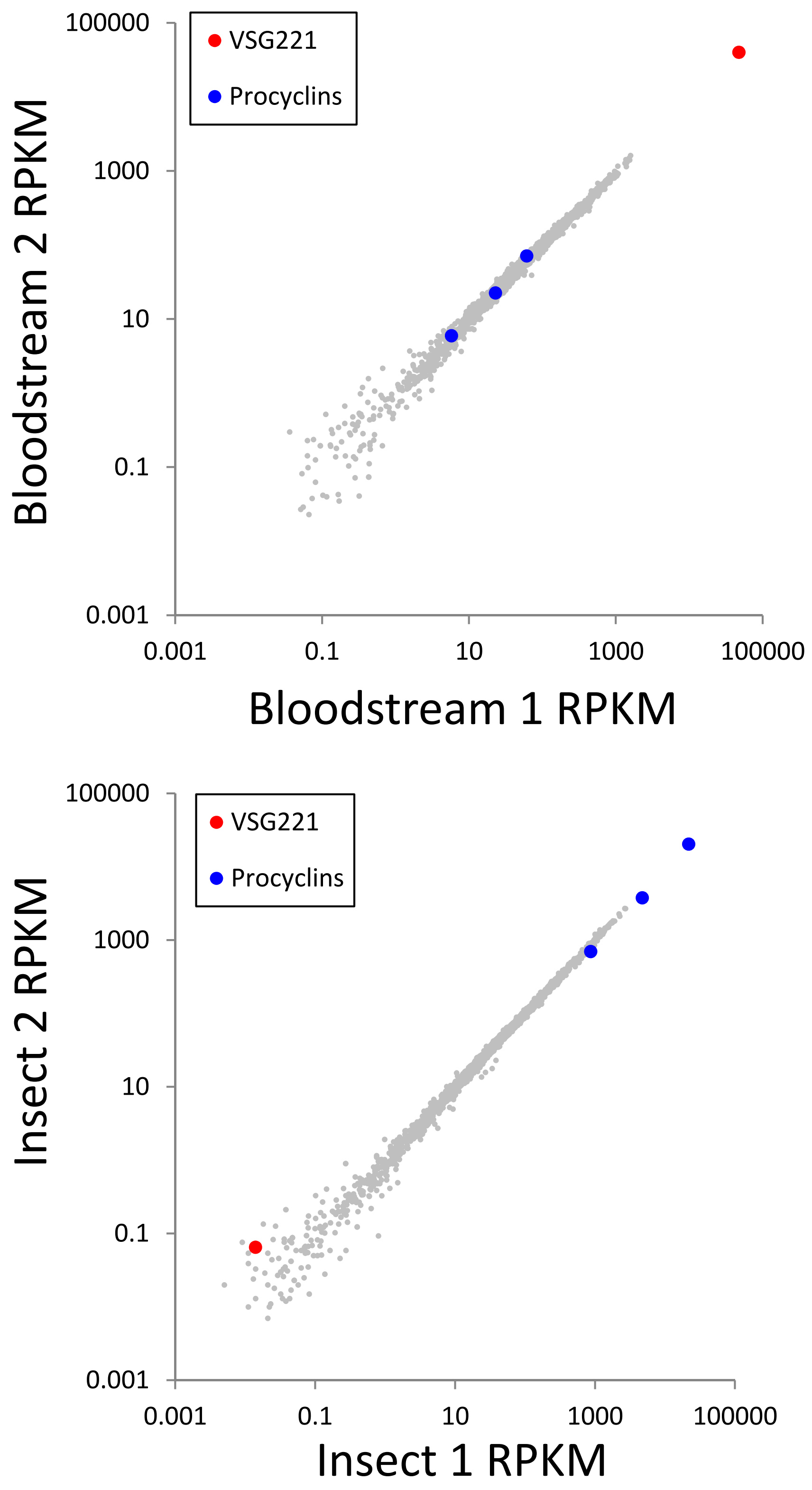

Additional file 2: Comparison of RNA-seq replicates. Scatter plots of bloodstream-form and insect-stage RPKM values for each condition. Data used to derive plots in Fig. 1 (MapQ > =0).
