# Supplementary material for: High-resolution analysis of multi-copy variant surface glycoprotein gene expression sites in African trypanosomes
Source: BMC Genomics. 2016 Oct 18;17:806. doi: 10.1186/s12864-016-3154-8 (PMC5070307; doi:10.1186/s12864-016-3154-8)
Supplement: Additional file 3: — ESAG7 sequences are highly similar between VSG-ESs. Clustal alignment between ESAG7 nucleotide sequence in BES1 (Tb429.BES40.2) and other ESAG7 sequences. BES2, 4 and 8 have multiple ESAG7 genes. (PDF 238 kb) [file 12864_2016_3154_MOESM3_ESM.pdf]

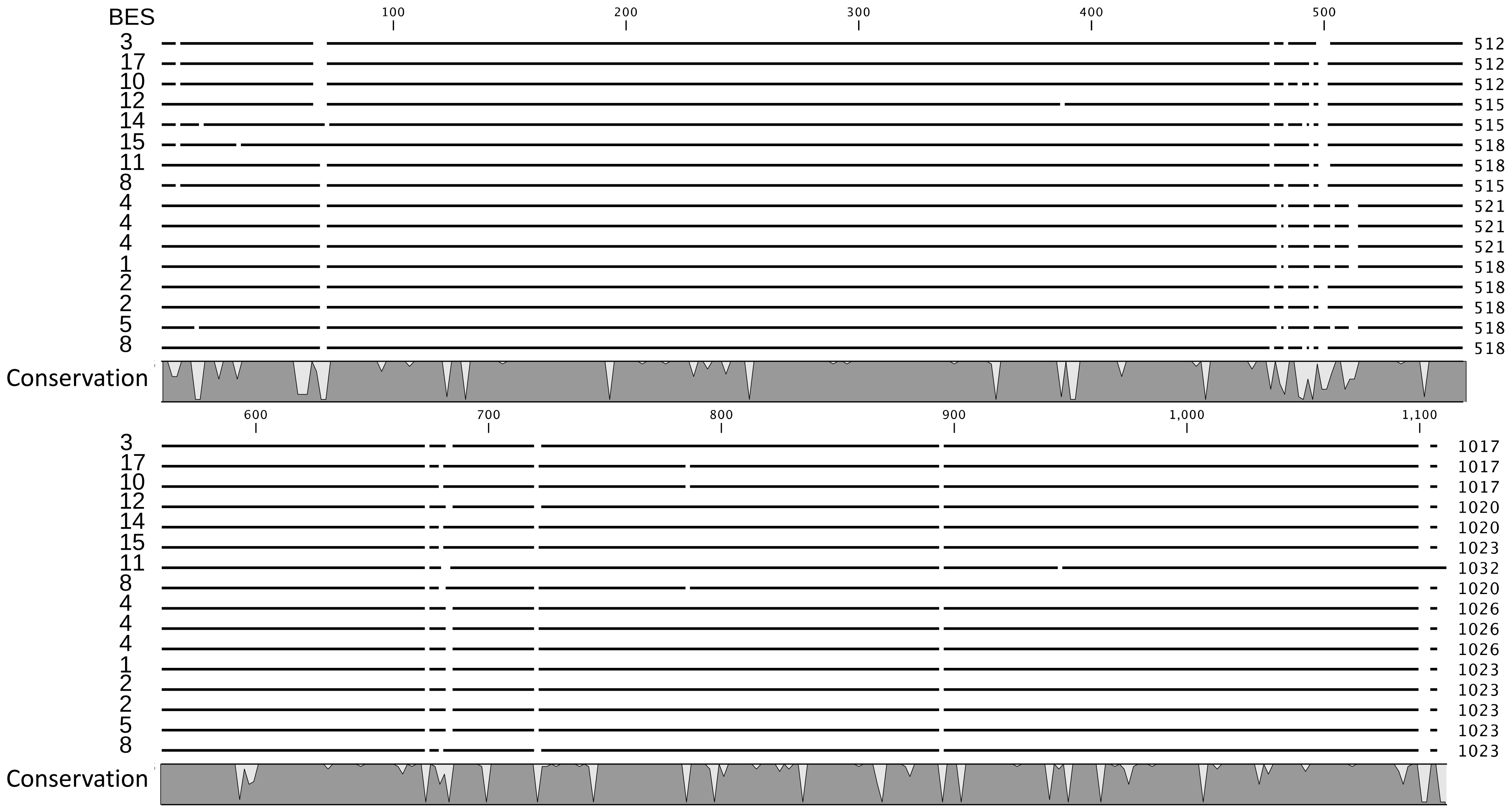

Additional file 3: *ESAG7* sequences are highly similar between *VSG*-ESs. Clustal alignment between *ESAG7* nucleotide sequence in BES1 (Tb429.BES40.2) and other *ESAG7* sequences. BES2, 4 and 8 have multiple *ESAG7* genes.
