# Supplementary material for: High-resolution analysis of multi-copy variant surface glycoprotein gene expression sites in African trypanosomes
Source: BMC Genomics. 2016 Oct 18;17:806. doi: 10.1186/s12864-016-3154-8 (PMC5070307; doi:10.1186/s12864-016-3154-8)
Supplement: Additional file 4: — Divergence increases towards the telomere. A. RPKM values for each ESAG and VSG in the non-redundant set plotted against the distance from the start-codon to the telomere. B. ESAG7 Clustal alignment plot from Additional file 3 compared to ESAG1 Clustal alignment plot. (PDF 169 kb) [file 12864_2016_3154_MOESM4_ESM.pdf]

A.

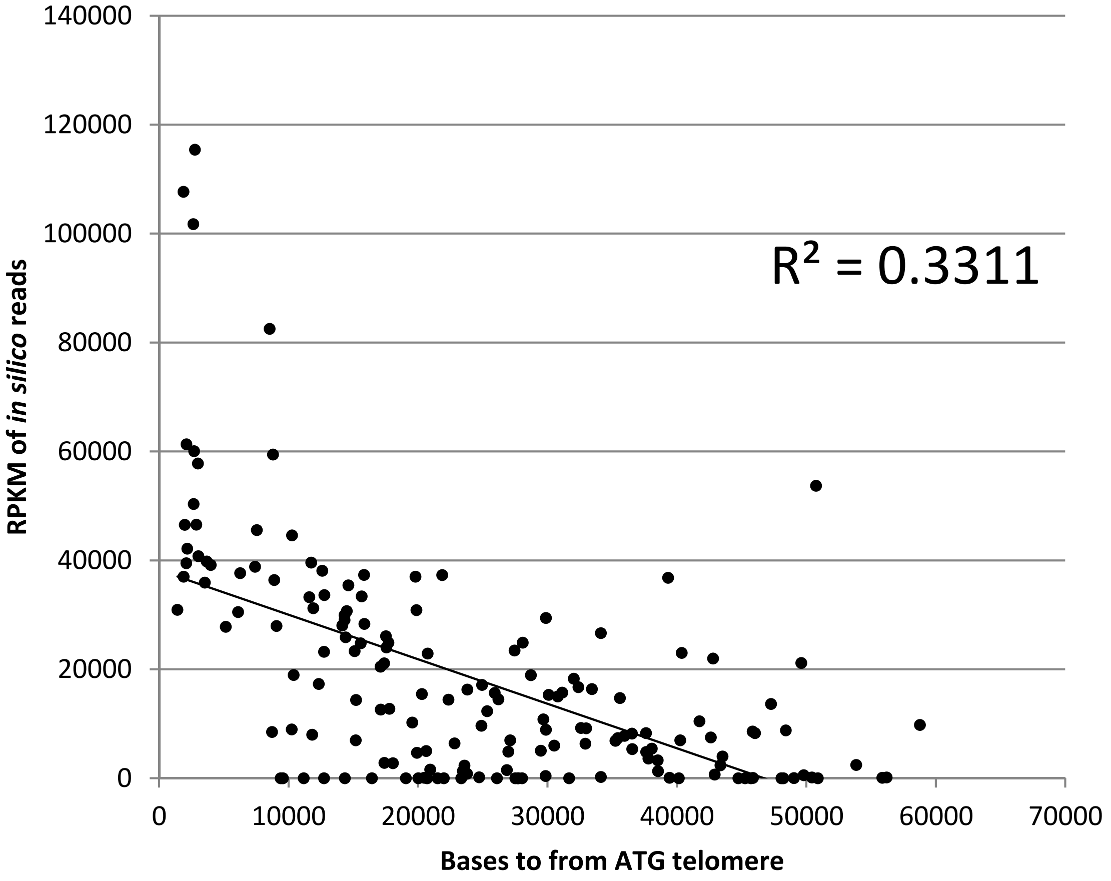

B.

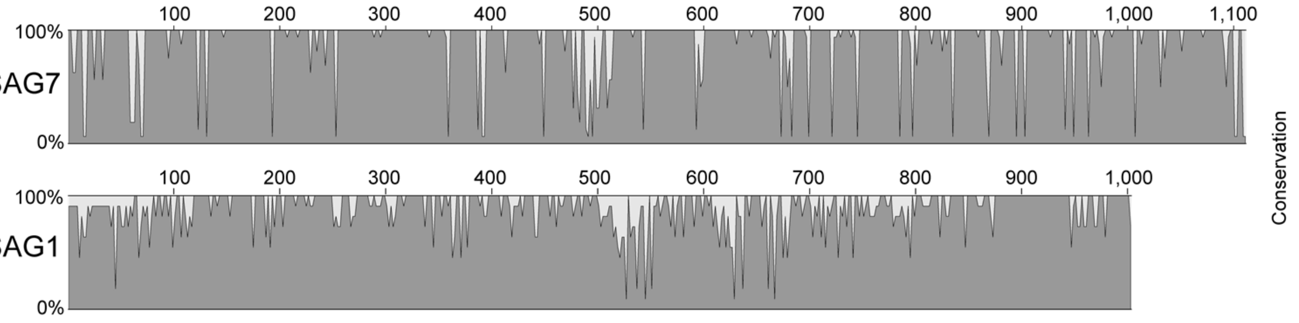

Additional file 4: Divergence increases towards the telomere. **A.** RPKM values for each *ESAG* and *VSG* in the non-redundant set plotted against the distance from the start-codon to the telomere. **B.** *ESAG7* Clustal alignment plot from Additional file 3 compared to ESAG1 Clustal alignment plot.
