# Supplementary material for: High-resolution analysis of multi-copy variant surface glycoprotein gene expression sites in African trypanosomes
Source: BMC Genomics. 2016 Oct 18;17:806. doi: 10.1186/s12864-016-3154-8 (PMC5070307; doi:10.1186/s12864-016-3154-8)
Supplement: Additional file 5: — High stringency RNA-seq analysis. Scatter plot of RPKM values of each condition with MapQ value > 1. Values are calculated as RPKM (Reads Per Kilobase of transcript per Million mapped reads) and are averages for a pair of independent sub-clones. Selected developmentally regulated genes are highlighted. Metacyclic (mVSG) and bloodstream (bVSG) specific VSGs are also shown. (PDF 23 kb) [file 12864_2016_3154_MOESM5_ESM.pdf]

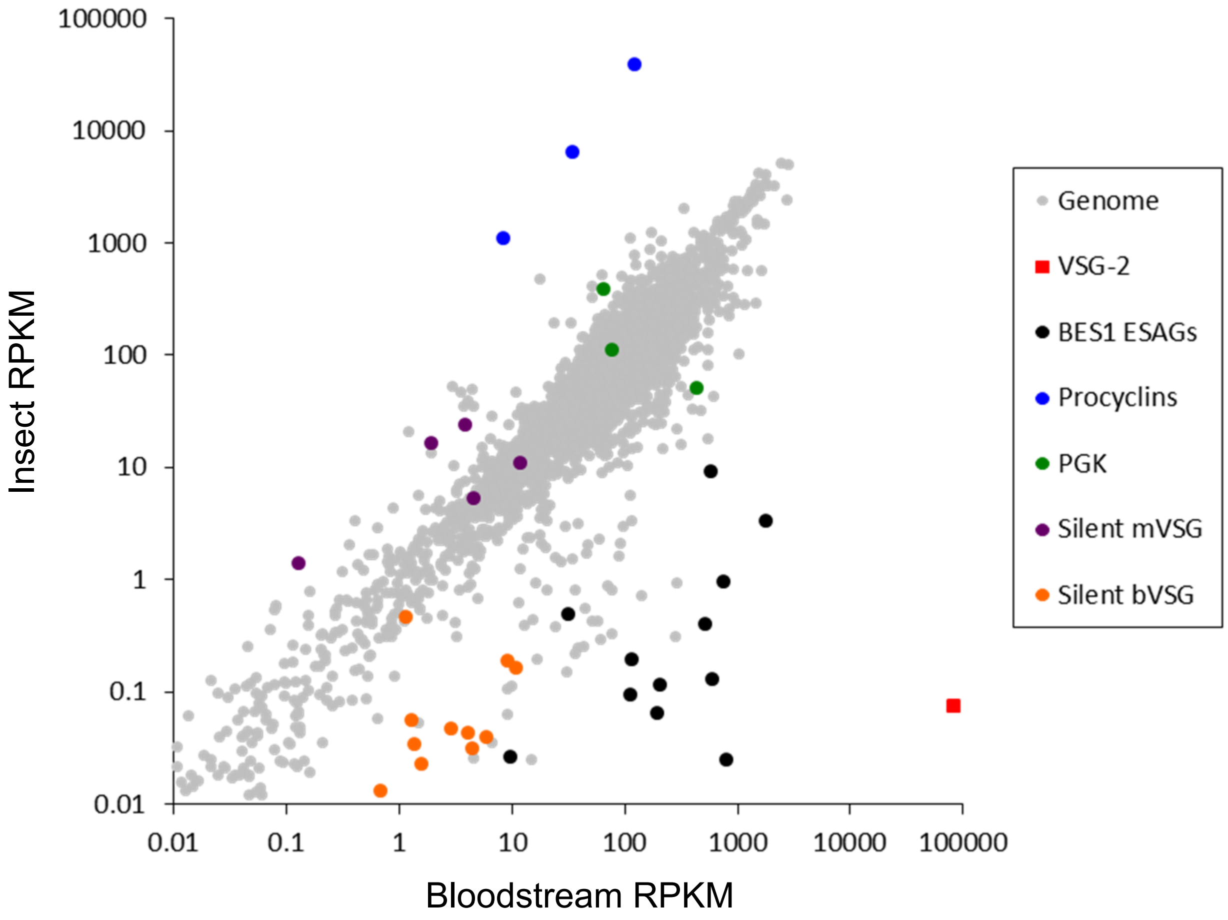

Additional file 5: High stringency RNA-seq analysis. Scatter plot of RPKM values of each condition with MapQ value > 1. Values are calculated as RPKM (Reads Per Kilobase of transcript per Million mapped reads) and are averages for a pair of independent sub-clones. Selected developmentally regulated genes are highlighted. Metacyclic (*mVSG*) and bloodstream (*bVSG*) specific *VSGs* are also shown.
