# Supplementary material for: High-resolution analysis of multi-copy variant surface glycoprotein gene expression sites in African trypanosomes
Source: BMC Genomics. 2016 Oct 18;17:806. doi: 10.1186/s12864-016-3154-8 (PMC5070307; doi:10.1186/s12864-016-3154-8)
Supplement: Additional file 6: — VSG genes have significantly shorter polypyrimidine tracts upstream. A. Logo plots of the 17 VSG trans-splice sites identified from our data set (top) and genes in the RNA Pol-I transcribed procyclin loci. Pyrimidines, red; purines, grey. B. Individual trans-slice sites from beta-tubulin (top), bloodstream VSG-ES linked VSGs and metacyclic VSGs. Pyrimidines, red; purines, grey. C. Bar charts show average polypyrimidine tract length comparisons over several gene groups. ***, P <0.0005. (PDF 1442 kb) [file 12864_2016_3154_MOESM6_ESM.pdf]

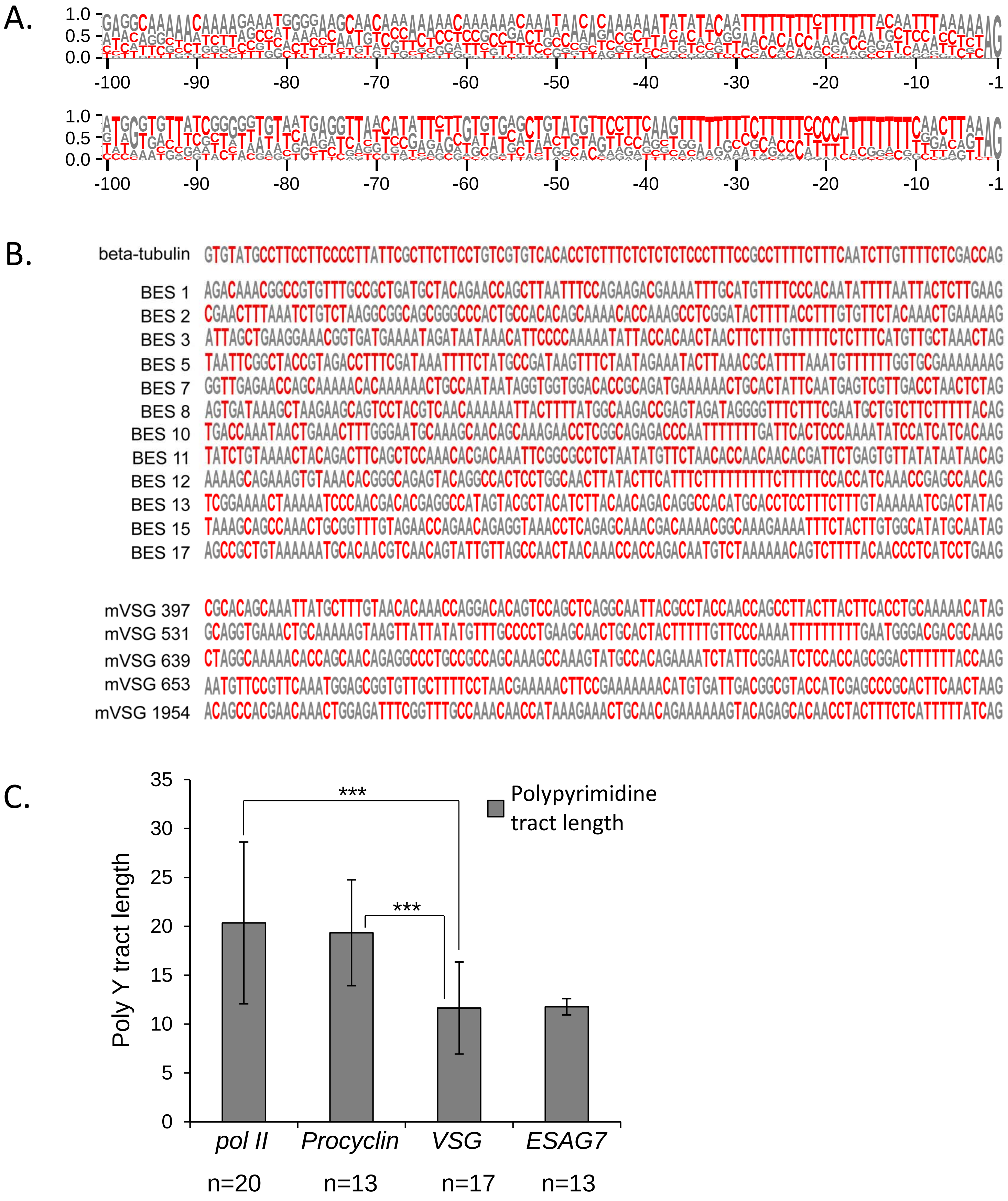

Additional file 6: *VSG* genes have significantly shorter polypyrimidine tracts upstream. **A.** Logo plots of the 17 *VSG trans*-splice sites identified from our data set (top) and genes in the RNA Pol-I transcribed *procyclin* loci. Pyrimidines, red; purines, grey. **B.** Individual *trans*-slice sites from beta-tubulin (top), bloodstream *VSG*-ES linked *VSGs* and metacyclic *VSGs.* Pyrimidines, red; purines, grey. **C.** Bar charts show average polypyrimidine tract length comparisons over several gene groups. ***, *P* <0.0005.
