# Supplementary material for: High-resolution analysis of multi-copy variant surface glycoprotein gene expression sites in African trypanosomes
Source: BMC Genomics. 2016 Oct 18;17:806. doi: 10.1186/s12864-016-3154-8 (PMC5070307; doi:10.1186/s12864-016-3154-8)
Supplement: Additional file 7: — Individual VSG-ES transcriptome analysis following VEX1 perturbation. Bar-charts show fold-changes for ESAG expression in each VSG-ES following either VEX1 knockdown (blue bars) or overexpression (red bars). (PDF 32 kb) [file 12864_2016_3154_MOESM7_ESM.pdf]

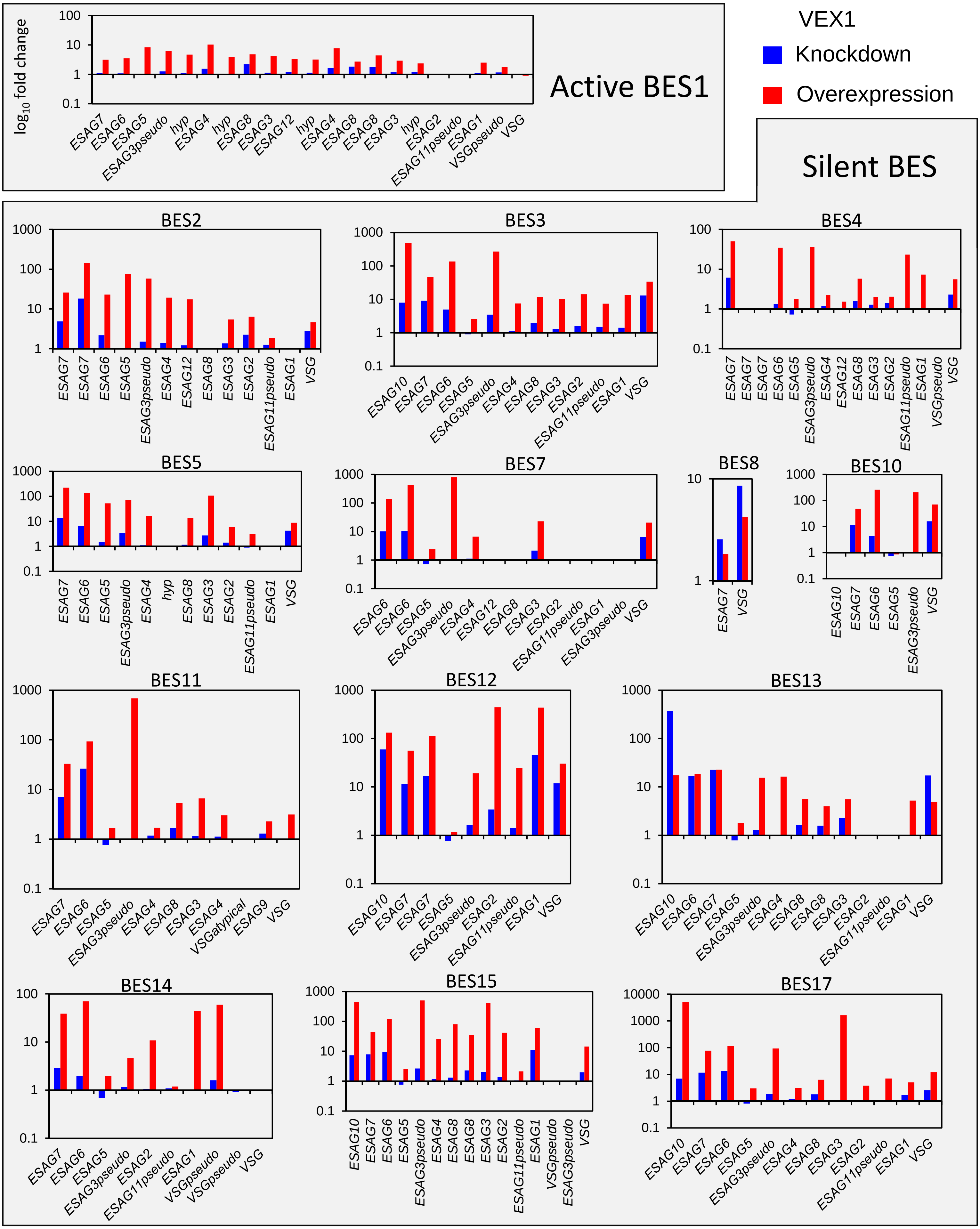


**Additional file 7:** Individual *VSG*-ES transcriptome analysis following VEX1 perturbation. Bar-charts show fold-changes for *ESAG* expression in each *VSG*-ES following either VEX1 knockdown (blue bars) or overexpression (red bars).
